# Optimizing consumer acceptability of 100% chocolate through roasting treatments and effects on bitterness and other important sensory characteristics

**DOI:** 10.1016/j.crfs.2022.01.005

**Published:** 2022-01-10

**Authors:** Alan P. McClure, Helene Hopfer, Ingolf U. Grün

**Affiliations:** a Patric Chocolate, 6601 Stephens Station Rd, Ste 109, Columbia, MO, 65202, USA; b Department of Food Science, The Pennsylvania State University, University Park, PA, 16802, USA; c Department of Food Science, University of Missouri, Columbia, MO, 65211, USA

**Keywords:** Cacao, Roasting, Chocolate, Bitterness, Liking, Consumer evaluation

## Abstract

Chocolate is a highly appreciated food around the world which is rich in polyphenols but usually sweetened to mask inherent bitterness and astringency. Here we aim to determine how roast time and temperature in cacao roasting affect bitterness intensity and consumer liking of chocolate. We have also determined the relationship between consumer liking and perceived bitterness, astringency, sourness, sweetness, and cocoa intensity. Unroasted cacao from three different origins was roasted according to a designed experiment into a total of 27 treatments which were evaluated for overall liking and sensory attribute intensities by 145 chocolate consumers. We demonstrate that bitterness, sourness and astringency of 100% chocolate can be reduced through optimizing roasting temperature and time. Reduction of bitterness, sourness and astringency were significantly correlated with increased acceptability of the unsweetened chocolate samples. Aside from roasting, cacao origin including base levels of bitterness, astringency, and sourness should also be considered when optimizing consumer acceptability. Perceived cocoa flavor intensity, being highly positively correlated to liking, is likely to also be an important consideration for raw material selection. As for optimal roast profiles, for the cacao origins in our study, more intense roasting conditions such as 20 min at 171 °C, 80 min at 135 °C, and 54 min at 151 °C, all led to the most acceptable unsweetened chocolate. Conversely, for the purposes of optimizing consumer acceptability, our data *do not* support the use of raw or lightly roasted cacao, such as 0 min at 24 °C, 11 min at 105 °C, or 55 min at 64 °C.

## Highlights

• Bitterness and astringency in chocolate can be reduced through optimizing roasting.
• Bitterness, sourness, and astringency are negatively correlated to consumer liking.
• Sweetness and cocoa intensity are correlated with increased liking of chocolate.
• Generally, more intense roasting conditions lead to more acceptable chocolate.
• Use of raw or lightly roasted cacao leads to less acceptable chocolate.

## Graphical abstract

**Figure:**
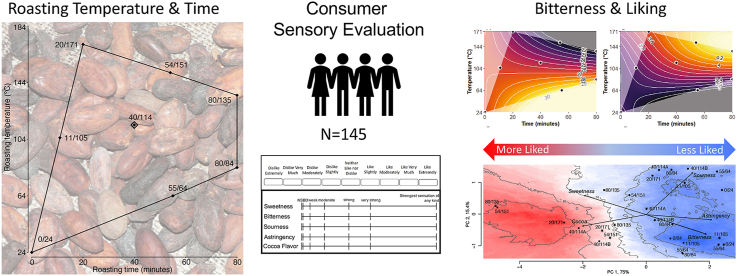

## 1 Introduction

Chocolate is a usually sweetened, solid paste that melts smoothly at human body temperature due to the presence and unique fatty acid composition of cacao fat, called cocoa butter (Aprotosoaie et al., 2016). Chocolate's unique flavor is due to compounds, such as flavonoids, methylxanthines, and Maillard-reaction products (Afoakwa et al., 2008). Also known as cocoa, cacao consists of the fermented and dried seeds of the tropical *Theobroma cacao* tree in the *Malvaceae* family (Aprotosoaie et al., 2016). Cacao is a significant food commodity, with annual global consumption reaching approximately 4.6 million metric tons as of 2018 with an increase in demand of 3.9% over 2017 (Barchart, 2019). Prior to transformation into chocolate, cacao is roasted to obtain more complex flavor and sensory characteristics that are preferred by consumers over those of raw cacao, e.g., lower bitterness (Aprotosoaie et al., 2016).

Bitterness, which is one of the five taste modalities (i.e., salty, sweet, sour, bitter, umami) sensed on the tongue (Gaudette and Pickering, 2013; Keast and Breslin, 2003), is generally disliked by humans (Drewnowski and Gomez-Carneros, 2000; Fischer et al., 2005) and even rejected in most foods (Gaudette and Pickering, 2013), which is potentially the result of evolution to detect bitter-tasting toxins (Keast et al., 2003). Famous exceptions to bitter food rejection are coffee, beer, red wine, and dark chocolate (Gaudette and Pickering, 2013; Keast and Breslin, 2003; Roy, 1997), which highlights the sometimes complex nature of human food choices (Gaudette and Pickering, 2013).

Of the basic tastes, bitter is the most complex (Drewnowski, 2001), with approximately 25 different subtypes of G-protein-coupled receptors called TAS2Rs which are responsible for the transduction of bitter taste from many thousands of compounds (Dagan-Wiener et al., 2018; Maehashi and Huang, 2009); these receptors are located in taste buds across the tongue, palate and throat (Drewnowski, 2001; Lawless and Heymann, 2010). Bitterness perception starts with the sensation of bitter compounds, but also includes processing by the brain of incoming signals from other sensory modalities (i.e., other tastes, aromas, and somatosensory, aural, and visual inputs) (Lawless and Heymann, 2010). For example, aural stimulation (i.e. music) was shown to affect bitterness perception (Carvalho et al., 2017).

In addition to genetic variation in bitter taste sensitivity (Drewnowski, 2001), sex- and age-based differences have also been described (Bartoshuk et al., 1994). Further, overall bitterness intensity of mixtures tend to be lower than the sum of intensities of the individual compounds at the same concentrations (Keast and Breslin, 2003), and bitterness may be suppressed by sweet, salty, and umami tastes, while being enhanced by sour (Calviño et al., 1993; Drewnowski, 2001; Fischer and Noble, 1994). Exposure to bitter compounds can lead to adaptation (i.e., decreased responsiveness) to bitterness (Lawless and Heymann, 2010), and bitter perception can also be altered by saliva components such as calcium ions (Neyraud and Dransfield, 2004) and proteins (Crawford and Running, 2020).

Bitter taste in cacao and chocolate specifically is thought to result predominantly from the presence of methylxanthines, such as theobromine and caffeine, and relatively low molecular weight flavonoids, including the flavan-3-ols epicatechin and its epimer catechin and some oligomers, as well as a variety of compounds in the 2,5-diketopiperazine (DKP) class (Stark et al., 2006). Some of these compounds are affected by cacao varietal, growing conditions, ripeness at harvest, and post-harvest processing such as fermentation and roasting ((Afoakwa et al., 2008; Aprotosoaie et al., 2016; Beckett et al., 2017; Kongor et al., 2016; Lemarcq et al., 2020). For example, geographical location, even within a single country, appears to affect methylxanthine concentration and theobromine to caffeine ratio (Carrillo et al., 2014). Roasting, considered by some to be the most important step in processing cacao (Aprotosoaie et al., 2016), results in the creation of bitter diketopiperazines (DKPs) from peptides (Rizzi, 1989; Ziegleder, 2017), and darker roasts, particularly at higher temperatures, appear to increase DKP levels the most (Bonvehí and Coll, 2000), whereas unroasted cocoa contains virtually no DKPs (Bonvehí and Coll, 2000). Roasting also alters the concentrations of the flavonoid epicatechin, and its epimers and oligomers (Kothe et al., 2013; Stanley et al., 2018), compounds which are both bitter and astringent (Stark et al., 2006), sometimes in unexpected ways that are varietal-specific (Kothe et al., 2013). Loss of epicatechin at temperatures over 70 °C occurs, and at a roasting temperature of 120 °C, catechin content has been seen to increase by approximately 650% in previously fermented cacao (Payne et al., 2010). Recently, McClure et al. (2021) also showed significant and large decreases in concentration of epicatechin and procyanidin B2 as roasting progressed, while at the same time significant increases were seen for catechin and cyclo(Proline-Valine).

Interestingly, our understanding of the variation of cacao-related bitterness, as can be surmised from the above-cited studies, has historically come mostly from instrumental investigation of the bitter compounds found in cacao (Lemarcq et al., 2021); the use of human sensory evaluation to understand such variation, on the other hand, has only slowly begun to gain favor in the 21st century, with most studies taking place only within the last 5 years (Lemarcq et al., 2021). Thus, we aim to close this gap by investigating consumer perception of bitterness and liking of chocolate made from cacao roasted with a variety of roast profiles, in order to better understand the impact of bitter perception on liking to the extent that optimizing consumer acceptability of 100% chocolate may be possible. We aimed to answer the following research questions:

1. What are the effects of roast time and roast temperature on perceived bitterness intensity and consumer liking ratings of chocolate?
2. What is the relationship between perceived bitterness and consumer liking ratings in chocolate, and are there roast-specific and/or origin-specific patterns underlying this relationship? Furthermore, do other measured sensory characteristics play an important role in understanding consumer liking as well?

## 2 Materials & Methods

The same materials and methods as previously reported (McClure et al., 2021) have been used; therefore, just a brief description is provided. For further details the reader is referred to McClure et al. (2021).

### 2.1 Materials

Three different lots of fermented and dried cacao, all falling within acceptable ranges for good fermented cacao according to the International Cocoa Organization ((ICCO), 2020) (i.e., less than 5% defective or slaty beans), from three origins (i.e., Madagascar, Ghana, Peru) and the 2018 and 2019 harvests, were obtained from Guittard Chocolate (Burlingame, CA), and Marañon Cacao (San Diego, CA). Prior to further processing, lots of each origin were composited, hand-sorted to remove dust, broken shell and beans, multiple bean clusters, unfilled beans, and foreign objects such as leaves, stones, or burlap twine, and stored in sealed Grainpro (Concord, MA) Supergrain Premium RT bags at <65% RH and <27 °C until roasted (approximately one month or less). Beans from all three origins were similar in size, ranging from 74 to 89 beans per 100 g. For the sensory training, aqueous solution (DI water) of food-grade tannic acid (41.5 g/L; Spectrum Chemicals, New Brunswick, NJ), citric acid (1.5 g/L; Sigma-Aldrich, St. Louis, MO), caffeine (1.0 g/L; Sigma-Aldrich), and sucrose (3.0 g/L; pure cane sugar, C&H, Crockett, CA) were used.

### 2.2 Methods

#### 2.2.1 Roasting experimental design

**Fig. 1:**
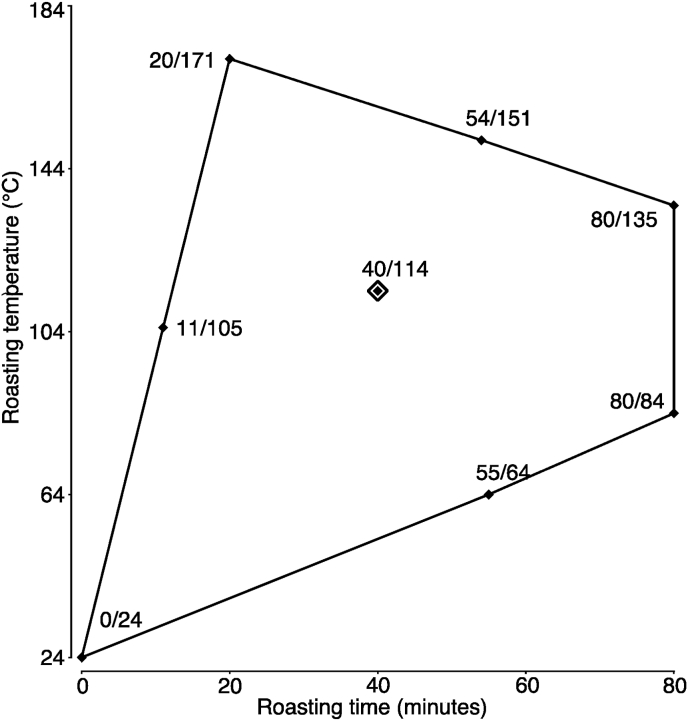
Modified I-Optimal experimental design for the 8 roasting treatments (duplicated center point at 40 min at 114 °C), shown as diamonds and labeled with their roasting time (in min) and temperature values (in °C), which were replicated for the three cacao origins. The total of 27 treatments were roasted in randomized order.

The roasting experimental design space (Fig. 1), with a temperature range from 24 °C to 171 °C and a time range from 0 to 80 min, was chosen based on literature (Afoakwa et al., 2008; Ziegleder, 2017) and feedback from chocolate professionals, and included a “raw” treatment at 24 °C (approximate room temperature) for 0 min as a control, excluding all impossible or extreme or repeat combinations of time and temperature (e.g., 80 min at 24 °C). A Response Surface Methodology (RSM) approach (JMP 14.0.0 software (Cary, NC)) was combined with an I-Optimal algorithm to minimize average variance of prediction for model coefficients (Jones and Goos, 2012; Myers et al., 2016; Oyejola and Nwanya, 2015), while at the same time minimizing covariance of model coefficients (Oyejola and Nwanya, 2015). The resulting design is an irregularly shaped non-rotatable design, related to a central composite design (CCD), with a duplicated centerpoint to allow for pure error estimation, similar to prior studies on roasting optimization (Farah and Zaibunnisa, 2012; Kahyaoglu, 2008; Lee et al., 2001; Madihah et al., 2012; Mendes et al., 2001; Özdemir and Devres, 2000). All time and temperature combinations were repeated for all three origins (Madagascar, Ghana, and Peru).

#### 2.2.2 Roasting and winnowing

All samples were roasted in a humidity and temperature-controlled environment using a forced air convection laboratory oven (model # FD56, Binder GmbH, Tuttlingen, Germany). For roasting and winnowing, two stainless steel mesh roasting trays were loaded with a single layer of 410 g of cacao and roasted for the required time in the oven after the oven reached the setpoint temperature and equilibrated for 10 min. Roasted cacao was cooled with a box fan (Lasko, West Chester, PA), and cracked with a CrankandStein (Atlanta, GA) 305 mm 3-roll cocoa cracker, and immediately winnowed with a custom food-grade winnower to remove shell and expedite cooling. Each roast was completed in duplicate on the same day, and nibs from all duplicates were blended until homogeneous, stored at less than 19.5 °C with relative humidity (RH) at approximately 40% or less and turned into chocolate liquor within 48 h.

#### 2.2.3 Chocolate liquor production

For chocolate liquor production, a Spectra 11 (Tamil Nadu, India) Stone Wet Grinder was used. After preheating the stone bowl and grinding stones to approximately 50 °C, the machine was then turned on and cacao nibs (1000 g) were added slowly over a period of 20 min. Once all nibs were added, the wet grinder was scraped four times in 30-min intervals, and chocolate liquor was refined to a smooth texture over 8 h. The final chocolate liquor was then poured through a Kitchenaid (Benton Harbor, MI) fine-mesh strainer into a plastic food-storage container, covered with an air-tight lid and stored at or below 19.5 °C and 40% RH or less. Solidified chocolate liquor batches were wrapped individually in aluminum foil, vacuum sealed in a multi-layer vacuum bag (FoodSaver, Oklahoma City, OK) with nylon vapor barrier, and stored at or below 19.5 °C with RH at approximately 40% or less until sensory evaluation (i.e., within 90 days).

Two days prior to sensory evaluation, the chocolate liquor samples were melted in wide-mouth glass jars, closed with aluminum foil at around 40 °C. Portioning of chocolate liquor was carried out in a humidity and temperature-controlled kitchen (44% RH, 23 °C), using positive displacement pipettes (Eppendorf, Hamburg, Germany), with pipettes set to 300 μL to obtain equal sample quantities (ca. 0.3 g) in the shape of small chocolate disks. Chocolate discs were deposited on parchment paper, and chilled in a commercial refrigerator (TRUE Manufacturing, O'Fallon, MO) set to 4 °C for approximately 1 h until the chocolate liquor disks had solidified. The chocolate disks were then transferred to labeled stainless steel food storage containers covered with aluminum foil prior to being returned to the refrigerator to maintain the texture of the disks until testing. This ensured a consistent mouthfeel and appearance, similar to that of tempered chocolate (i.e., gloss and absence of bloom and grittiness). All 27 samples were prepared in this way over the course of approximately 36 h.

#### 2.2.4 Sensory evaluation

For sensory evaluation of the 27 chocolate liquor samples (i.e., all treatments across all 3 origins of cacao) 145 consumers (aged 18–65, 38 males) were recruited from the in-house database of the Sensory Evaluation Center (SEC) in the Food Science department at The Pennsylvania State University, University Park, PA, based on the following screening criteria: between 18 and 65 years of age, no food allergies or sensitivities, no taste or smell deficiencies or difficulties to swallow, no mouth piercings, not taking any medications, non-smokers, neither pregnant nor breastfeeding, regular consumers of chocolate products (at least 1x/month), and a preference for either milk or dark chocolate. Informed implied consent was obtained at the beginning of the recruitment screener and sensory test, and research procedures were deemed exempt from institutional review board overview by the Penn State Office of Research Protections under the wholesome foods exemption in 45 CFR 46.101(b) (protocol number 33164). Consumer participants were compensated for their time according to the IRB protocol.

Consumers were asked to come to the SEC on five consecutive days, evaluating 5 different samples each day, according to a modified incomplete Williams Latin square design to control for first-order carryover effects, and to ensure that each sample was evaluated an average of 148 times by an average of 104 consumers. On the first testing day, each consumer completed a brief sensory training (Hamada et al., 2020), both to familiarize participants with the attributes to be rated, as well as how to use the Generalized Labeled Magnitude Scale (gLMS). Participants were presented with the five samples served on individual tasting spoons placed on a serving tray with a tray mat to indicate the evaluation order and instructed to place the entire sample disk in their mouth. They first rated overall liking on a 9-point hedonic scale, followed by rating the perceived intensities for the attributes astringent, sour, bitter, sweet, and cocoa/dark chocolate (i.e., *The intensity and richness of deep dark chocolate and cocoa flavors. For example, a piece of dark chocolate, or the smell of freshly baked chocolate brownies*). During the mandatory 2-min break in between samples, participants were asked to cleanse their palate with room temperature reverse osmosis water. Finally, data on chocolate preference, chocolate consumption frequency, gender, age, and ethnicity were collected after the last sample assessment.

### 2.3 Data analysis

Analysis of the sensory data took place in RStudio (v. 1.2.1334) running R version 3.6.0. Packages used include lmerTest (v. 3.1–2: Kuznetsova et al., 2017) for mixed-model selection and analysis, MuMIn (v.1.43.17: Barton, 2020) for model pseudo R^2^ calculation, ggplot2 (v.3.3.2: Wickham, 2016) for contour plots, and FactoMineR (v. 2.3: Lê et al., 2008) and SensoMineR (v.1.26: Lê and Husson, 2008) for preference maps.

For the consumer data, mixed-effects linear regression models were fit to the data for both Bitterness and Liking separately as the response variables, with the fixed variables: time, temperature, origin, and all their two and three-way interactions and the random variables: consumer, evaluation day, sample order across all five days (1–27), sample order within evaluation day (1–5), age group (5 bins between 18 and 65 years), chocolate preference, and the consumer-by-day interaction. Time and Temperature were mean-centered and scaled prior to analysis. Origin is a categorical variable designating cacao from three specific geographical locations (see Materials & Methods).

In model diagnostic plots, residuals were found to have a non-skewed distribution with some lack of normality in the tails, and a square-root transformation to the response substantially corrected the issue. After transformation, backward stepwise model selection was performed to find the most parsimonious fixed effects model while maintaining only significant random effects terms. Variance inflation factor (VIF) tests for selected models showed that VIFs were less than ∼2.5 for all first order main effects and generally all effects, ruling out multicollinearity as an important contributor to coefficient estimate error and term significance. Type III ANOVA for unbalanced data was performed to obtain estimates of p-values and coefficients for each term in the model. Given the presence of random effects, pseudo R^2^ values were instead computed, and were all greater than 0.6. Given the nature of the data (i.e., psychophysical data based upon sensory analysis), R^2^ values over 0.25 are considered large effects (Cohen, 1988; Hemphill, 2003). Additionally, contour plots were prepared for each model to visualize the predicted values of the selected models.

## 3 Results

### 3.1 Bitterness perception as a function of roasting conditions and cacao origin

**Fig. 2:**
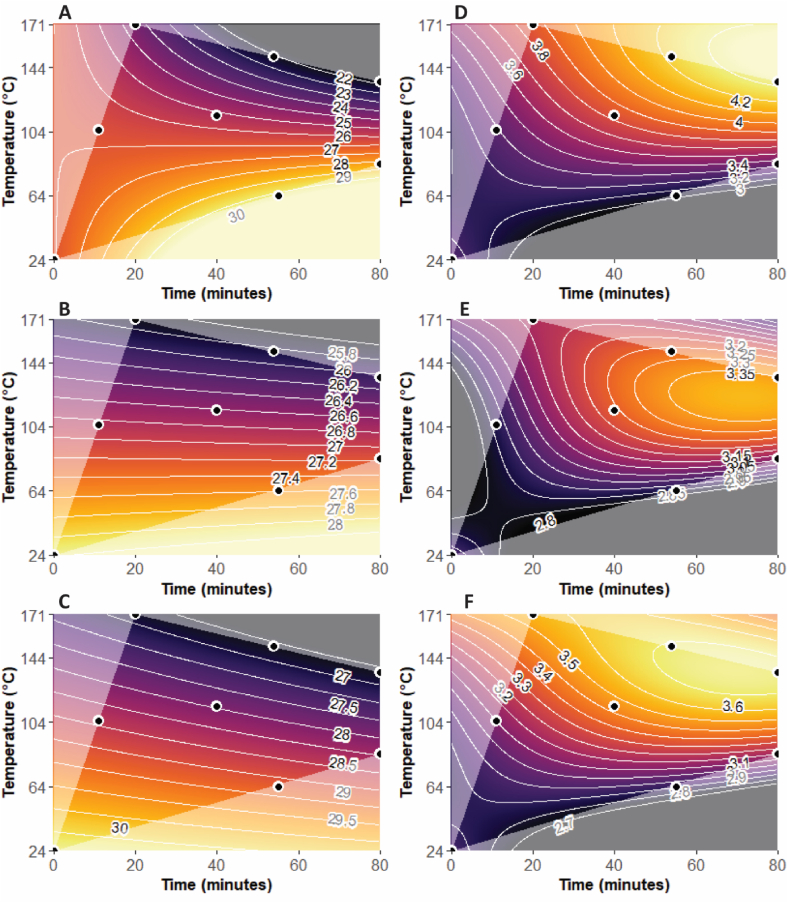
Surface-response plots for (**A-C**) Perceived Bitterness, and (**D-F**) Overall Liking of 100% chocolate across the entire experimental roasting region for (**A,D**) Ghana, (**B,E**) Madagascar, and (**C,F**) Peru. Individual roasts are indicated as black dots.

The focus of this study was to understand how bitterness varies with roasting, with a particular focus on how it decreases in relation to roasting time and temperature. The change of bitterness with roasting for each origin is visualized in Fig. 2A–C. The mean values of bitterness for the three origins fell between moderate (=17) and strong (=35) in intensity on the gLMS scale. Still, there were some significant differences in mean bitterness between the origins, with Peru (mean bitterness = 28.1) being significantly more bitter than both Ghana (mean = 26.0, *p* = 0.0001) and Madagascar (mean = 26.8, *p* = 0.016); there was no significant difference in mean bitterness between Ghana and Madagascar (*p* = 0.13). Despite the previously noted differences in mean values, the patterns of bitterness change for each origin show certain similarities, with highest bitterness intensity in the lower half of the region (i.e., between the raw treatment (0 min/24 °C) and the lightly roasted treatment (55 min/64 °C)) (Fig. 2A–C). Additionally, the overall direction of bitterness decrease from the lower half to the upper half of each plot is also similar for all origins, meaning that the estimated region of lowest bitterness for each origin always falls between 135 °C and 171 °C, with lowest achievable bitterness of 22, 25.8, and 27 for Ghana, Madagascar and Peru, respectively, on the gLMS scale (i.e., between moderate (=17) and strong (=35)).

However, it is interesting that across the entire experimental region, overall perceived bitterness decreased more for Ghana (from 30 to 22, or 8 points on the gLMS scale), than it did for Madagascar (28.2–25.8, only a 2.4 point decrease) or for Peru (30–27, only a 3 point decrease). This means that overall, roasting had a greater than 2-fold larger impact on perceived bitterness decrease for Ghana compared to the other two origins.

Given that more roasting (i.e., higher temperatures and longer time) resulted in less bitterness for all origins, the most important terms in the model for understanding bitterness change due to roasting in this study were roasting time, roasting temperature, and their interaction term (Table 1A). Comparing the coefficient estimates for the bitterness intensity model (pseudo R^2^ of 65.6%; Table 1A), roasting temperature had the largest effect, with an increase in roasting temperature by one standard deviation (or 42 °C) leading to a decrease in the mean value of the square root of bitterness intensity (i.e., 5.2) by 0.153 units, which is equivalent to a decrease in the mean value of bitterness (i.e., 26.9) of 1.56 units on the gLMS scale. In contrast, the time-temperature interaction had about half of that effect and roasting time had about a fifth of this effect. This is also visualized in Fig. 2A–C, where for each origin, bitterness intensity dropped more with increasing roasting temperature compared to roasting time.

**Table 1:** Coefficient estimates and pseudo R^2^ values for the **(A)** Bitterness and **(B)** overall Liking regression models. Coefficient estimates are reported as the change in the square-root transformation of the response per 1 standard deviation increase (i.e., 27 min or 42 °C) in roasting time and roasting temperature, respectively.

	(A)	(B)
	Bitterness	Liking
Time	−0.02853	5.938e-02*
Temperature	−0.15290*	9.046e-02*
Time:Temperature	−0.06640*	3.763e-02*
Time^2^	–	−2.459e-02*
Temperature^2^	–	−4.309e-02*
Time: Temperature^2^	–	−4.382e-02*
Temperature:Time^2^	–	–
Pseudo R^2^	65.6%	62.5%

* model term is significant (*p* ≤ 0.05).

It is not clear why the Ghana sample plot (Fig. 2A) shows more curvature compared to the other two origins Madagascar (Fig. 2B) and Peru (Fig. 2C). It could be the Ghana samples cover a wider range of changes due to roasting compared to the other two origins which would also explain the larger range of bitterness intensity in the Ghana samples.

### 3.2 Consumer liking as a function of roasting conditions and cacao origin

We also modelled how consumer liking varies with roasting, with a particular focus on how consumer acceptability changes as a function of roasting time and temperature. The change of liking with roasting for each origin is visualized in Fig. 2D–F. The mean values of liking for the origins all fell between dislike moderately (=3) and dislike slightly (=4) on the 9-point hedonic scale. This is an expected range of liking scores, given the unsweetened nature of all samples. Still, there were significant differences in mean liking between the origins, with Ghana (mean liking = 3.7) being significantly more liked than both Peru (mean = 3.4, *p* = 1.076 × 10^−8^) and Madagascar (mean = 3.2, *p* < 2.2 × 10^−16^); Peru was also better liked than Madagascar (*p* = 0.0005). Despite these differences in mean values, the patterns of liking change for each origin are relatively similar, with maximal liking in the upper-right portion of the experimental region for each origin, i.e., between the roasted treatments (54 min/151 °C) and (80 min/135 °C) (Fig. 2D–F). The overall direction of liking increases from the lower left quadrant to the upper right quadrant of each plot similarly for all three origins, with highest achievable liking scores of 4.3, 3.4, and 3.7 for Ghana, Madagascar and Peru, respectively (i.e., on the 9-point scale arranged between dislike moderately (=3), dislike slightly (=4), and neither like nor dislike (=5)). However, it is interesting that across the entire experimental region, perceived liking increased more overall for Ghana (from 3.0 to 4.3, or 1.3 points on the 9-point scale) and Peru (2.7–3.7, a 1-point increase) than it did for Madagascar (2.8–3.4, a 0.6-point increase). This means that overall, roasting increased liking approximately 2-fold more for Ghana and Peru than for Madagascar.

Still, given that more roasting resulted in increased liking for all origins, the most important terms in the model for understanding liking change in this study were therefore roasting time, roasting temperature, and their interaction terms (Table 1B). Comparing the coefficient estimates for the liking intensity model (pseudo R^2^ of 62.5%; Table 1B), roasting temperature has the largest effect, with an increase in roasting temperature by one standard deviation (or 42 °C) leading to an increase in the mean value of the square root of liking (i.e., 1.86) by 0.091 units, which is equivalent to an increase in the mean value of liking (i.e., 3.46) by 0.34 units on the 9-point hedonic scale, while time had just over half of that effect and the time-temperature interaction had just under half of this effect. Additional terms added significant curvature to the model. This is all visualized in Fig. 2D–F. These figures also make it clear that the experimental region includes a probable region of optimal liking for both Peru and Madagascar, as an optimized region for consumer acceptability was identified (Fig. 2E–F). For the Ghana origin however, additional roasting treatments in the upper right quadrant would be needed to identify where the optimal roasting treatment for Ghana would lie as the optimum appears to lie outside of the experimental region (Fig. 2D). This would likely include combinations of higher roasting temperatures (e.g., 160 °C) and roasting times (e.g., 90 min).

### 3.3 Relationship of sensory characteristics to consumer liking ratings

**Fig. 3:**
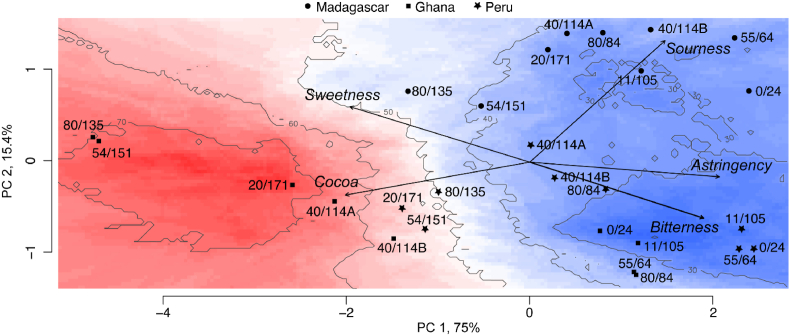
External Preference Map linking the sensory attributes of sweetness, sourness, astringency, bitterness, and cocoa flavor to liking of the 27 cocoa roasting treatments. Different origins are shown as different symbols followed by roasting time(min)/temperature(°C) treatment. Areas of high acceptability are shown in red shades located on the left hand side, and areas of low acceptability are shown in blue, on the right hand side. (For interpretation of the references to colour in this figure legend, the reader is referred to the Web version of this article.)

Bitterness and liking data were analyzed together to produce an External Preference Map (Fig. 3), the goal of which is to better understand consumer acceptability of various treatments in relation to their consumer-rated sensory characteristics (i.e., bitterness, astringency, sourness, cocoa, sweetness) (Lê and Worch, 2014). The principal component regression (PCR) model on which this specific map is based has an adjusted R^2^ of 0.86 (*p* = 2.167 × 10^−11^), and predictors consist of the first two dimensions of the principal component analysis (PCA), which explain 90.4% of the variation. The preference map (Fig. 3) clearly shows a pattern of greater acceptability for treatments with more intense roasts, i.e., higher temperatures and longer times, with the least acceptable treatments, generally speaking, being either raw (i.e., 0 min/24 °C) or lightly roasted (e.g., 11 min/105 °C and 55 min/64 °C). In addition to these roast-profile-related liking patterns, there are also apparent origin-specific patterns in liking. The five most liked treatments, i.e., acceptable to more than 60% of consumers, are all from the Ghana origin, while four of the six least liked treatments, with acceptability of ∼30% or fewer consumers, are Peruvian. Most Madagascar treatments are spread in the low to middle range of 40–50% consumer acceptability, while Peruvian treatments range from the least liked (i.e., <30% acceptability) to just above 50% consumer acceptability. Ghana treatments clearly show the largest increase in consumer liking due to roasting treatment, ranging from less than 30% to over 70% acceptability, or a change in acceptability of greater than 40%.

On average, samples from Peru showed greater bitterness intensity than the other two origins, and Ghanaian treatments are least bitter overall. This is certainly one likely explanation for the lower acceptability of Peruvian and Madagascar treatments compared to the Ghanaian treatments. It is also apparent that the lower acceptability of these samples is a result of the higher levels of astringency and sourness, as both of these sensory attributes show a high correlation to bitterness intensity. On the other hand, cocoa flavor and sweetness intensity are both positively correlated with consumer acceptability.

## 4 Discussion & conclusion

### 4.1 Discussion

In our study, more roasting (i.e., higher temperatures and longer time) resulted in increased liking for all origins, with roasting temperature being a larger driver than roasting time, similar to findings by Rocha et al. (2017). This makes sense, as cacao is roasted to obtain a more complex flavor and sensory characteristics that are preferred by consumers over those of raw cacao (Aprotosoaie et al., 2016). Additionally, more roasting (i.e., higher temperatures and longer time) also resulted in decreased bitterness for all origins, which is supported by recent findings on these same cacao treatments—all three origins – where bitter and astringent compounds, such as the flavan-3-ols epicatechin and procyanidin B2, are both reduced substantially due to roast (McClure et al., 2021). Epicatechin, specifically, has been noted as one of the fundamental bitter compounds in cacao and chocolate (Stark et al., 2006), however, there are many more bitter-taste inducing compounds in chocolate (Afoakwa et al., 2008; Aprotosoaie et al., 2016; Ziegleder, 2017; Lemarcq et al., 2020). Altogether, this decrease in bitterness from roasting, as well as decreases in other sensory characteristics such as astringency and sourness, helps to explain why roasting is “crucial in profile development” of chocolate (Kauz et al., 2021) and quite important for understanding consumer acceptance.

Specifically, in our study, decreases in bitterness and astringency were correlated with increased consumer acceptability of samples. This pattern has been seen in previous sensory analysis of chocolate, with bitterness and astringency having been tied to significantly decreased consumer acceptance (Harwood et al., 2013), irrespective of self-reporting by consumers of chocolate preferences (e.g., dark or milk chocolate). This makes sense, because bitterness, is generally disliked by humans (Drewnowski and Gomez-Carneros, 2000; Fischer et al., 2005) a behavior which is potentially the result of evolution to detect bitter-tasting toxins (Keast et al., 2003). As might be expected, increases of cocoa intensity and sweetness are positively correlated with chocolate acceptability, and given that sweetness increases can result in suppression of characteristics such as bitterness and sourness, this is hardly surprising (Lawless and Heymann, 2010).

One limitation of our study is the analysis by chocolate consumers of unsweetened chocolate not containing any other ingredients. Additions of sugar, salt, additional cocoa butter and other ingredients would most likely change the sensory properties of the resulting chocolates and lead to mixture suppression and/or enhancement effects (Lawless and Heymann, 2010) that would be relevant for liking, especially given the correlation between sweetness and liking in chocolate noted in our models. Additionally, particularly in so called fine or flavor chocolate, other aroma notes, such as floral, fruity, or nutty, may be relevant to the liking ratings (Aprotosoaie et al., 2016) via e.g., cross-modal interactions (Noble 1996), which have not been studied here.

Among the strengths of our study is the use of a large number of actual chocolate consumers of all genders, ages, and ethnicities to evaluate these samples instead of relying on a trained panel. This means that our results are directly relatable to end chocolate consumers. Additionally, the use of an optimal experimental design (i.e., I-Optimal algorithm-selected combination of roasting times and temperatures from within the selected ranges for all origins), covering the range of industrially applied roasting treatments for 3 different cacao origins provide far-reaching and widely applicable results.

## 5 Conclusion

In this study we demonstrate that bitterness, sourness, and astringency of 100% chocolate can be reduced through optimizing roasting parameters, such as roasting temperature and time. In turn, reducing the perceived intensity of bitterness, sourness, and astringency was found to lead to increased acceptability of these unsweetened chocolate samples.

In addition, we also found that the raw material – here, the origin of the cacao beans, also contributes to the sensory perception of the chocolate. This means that selection of raw material naturally low in these characteristics, such as the Ghana sample in our study, combined with optimizing roast, would be the best overall approach to minimize bitter and sour taste and astringent mouthfeel in chocolate and subsequently, to increase liking and acceptability. Additionally, it is likely that consumers’ perception of sweetness and cocoa flavor both lead to increased acceptability, as the intensity of both were well correlated with consumer liking.

Although optimized roasting conditions for minimal bitterness and maximal liking varied somewhat between origins, and there is not a complete understanding of this variation within or between origins, it appears that in general, roasting conditions such as 20 min/171 °C, 80 min/135 °C, and 54 min/151 °C, lead to most acceptable unsweetened chocolate. Similarly, if maximizing acceptability is a consideration, our data do not support the use of raw or lightly roasted cacao, such as 0 min/24 °C, 11 min/105 °C, or 55 min/64 °C.

## Funding

This work was supported by a grant from the 10.13039/100004068Professional Manufacturing Confectioners Association (PMCA; project 00062604). The PMCA was not involved in study design, nor in the collection, analysis, and interpretation of data, and was not involved in the writing of the report, nor in the decision to submit this article for publication. Dr. Hopfer is supported by the USDA National Institute of Food and Agriculture Federal Appropriations under project PEN04624 and accession number 1013412.

## CRediT authorship contribution statement

**Alan P. McClure:** Conceptualization, Data curation, Formal analysis, Funding acquisition, Investigation, Methodology, Project administration, Resources, Supervision, Validation, Visualization, Writing – original draft, Writing - review & editing. **Helene Hopfer:** Conceptualization, Data curation, Investigation, Project administration, Resources, Software, Supervision, Writing – original draft, Writing - review & editing. **Ingolf U. Grün:** Funding acquisition, Investigation, Project administration, Resources, Supervision, Writing - review & editing.

## Declaration of competing interest

The authors declare the following financial interests/personal relationships which may be considered as potential competing interests: Alan McClure reports financial support was provided by Professional Manufacturing Confectioners Association (PMCA). Alan McClure reports a relationship with Patric Chocolate that includes: employment. Samples of cacao were received as donations from two chocolate companies, there was no specific expectation attached to such donations outside of sharing conclusions drawn from this study - APM In the last five years, Dr. Hopfer has consulted for for-profit food/consumer product corporations on projects wholly unrelated to this study. - HH Dr. Hopfer is also the Associate Director of the Sensory Evaluation Center at Penn State, which routinely conducts product tests for industrial clients to facilitate experiential learning for students. - HH.
